# Eagle-449: A volumetric, whole-brain compilation of brain atlases for vestibular functional MRI research

**DOI:** 10.1038/s41597-023-01938-1

**Published:** 2023-01-14

**Authors:** Jeremy L. Smith, Vishwadeep Ahluwalia, Russell K. Gore, Jason W. Allen

**Affiliations:** 1 Department of Radiology and Imaging Sciences, Emory University School of Medicine, Atlanta, Georgia USA; 2 Georgia Institute of Technology, Atlanta, Georgia USA; 3 GSU/GT Center for Advanced Brain Imaging, Atlanta, Georgia USA; 4 Wallace H. Coulter Department of Biomedical Engineering, Georgia Institute of Technology and Emory University, Atlanta, Georgia USA; 5 Shepherd Center, Atlanta, Georgia USA; 6 Department of Neurology, Emory University School of Medicine, Atlanta, Georgia USA

**Keywords:** Sensorimotor processing, Neurological disorders

## Abstract

Human vestibular processing involves distributed networks of cortical and subcortical regions which perform sensory and multimodal integrative functions. These functional hubs are also interconnected with areas subserving cognitive, affective, and body-representative domains. Analysis of these diverse components of the vestibular and vestibular-associated networks, and synthesis of their holistic functioning, is therefore vital to our understanding of the genesis of vestibular dysfunctions and aid treatment development. Novel neuroimaging methodologies, including functional and structural connectivity analyses, have provided important contributions in this area, but often require the use of atlases which are comprised of well-defined *a priori* regions of interest. Investigating vestibular dysfunction requires a more detailed atlas that encompasses cortical, subcortical, cerebellar, and brainstem regions. The present paper represents an effort to establish a compilation of existing, peer-reviewed brain atlases which collectively afford comprehensive coverage of these regions while explicitly focusing on vestibular substrates. It is expected that this compilation will be iteratively improved with additional contributions from researchers in the field.

## Background & Summary

Cortical areas responsive to vestibular stimuli were first delineated using visual and somatosensory stimuli as well as ligand-based tract-tracing in nonhuman primates. These areas include intraparietal area *2* *v*, a section of area 3a (denoted *3aV*), frontal area 6, superior parietal/supramarginal gyrus areas 7a and 7b,and a region, denoted parieto-insular vestibular cortex, or PIVC, which is located in the Sylvian fissure and is densely connected with these areas as well as the vestibular nuclei in the brainstem^1–5^. Eickhoff and colleagues^6,7^ conducted histological examinations of the parietal operculum and posterior insula in humans, and proposed that the parieto-insular region was comprised of four cytoarchitectonically distinct subregions: *OP1*, the equivalent of primate secondary somatosensory cortex; *OP2*, localized to the depths of the insular-retroinsular Sylvian fissure, and for which no primate equivalent was found; *OP3*, equivalent to primate somatosensory area VS; and *OP4*, equivalent to primate area PV. In conjunction with galvanic vestibular stimulation (GVS) studies, Eickhoff *et al*. proposed that OP2 might serve as the human homologue of the nonhuman primate PIVC. Zu Eulenberg and colleagues^8,9^ have since leveraged GVS and functional MRI (fMRI) to further localize human vestibular regions, confirming GVS-correlated responses in OP2, 2 v, 3aV, premotor area 6, and area 7 (designated as PF in the von Economo-Koskinas system^10–13^), as well as the cerebellar nodules and uvula, visual area *hMST*, parietal area *PFcm*, area *hIP3* of the intraparietal sulcus, and visual cingulate (*CSv*, caudal Brodmann area 23), equivalent to primate areas VPS, VIP, and vestibular cingulate, respectively, after controlling for somatosensory, nociceptive, and salience effects, and concluded that the “core” human vestibular network likely consists of OP2, hMST, PF and PFcm, and CSv. These areas were affirmed via tractography, GVS, and resting-state functional connectivity (RSFC) based methods by Raiser *et al*.^14^, who also noted that the connectivity of this network is interhemispheric, predominantly right-lateralized, and characterized by demonstrable RSFC between homotopic regions.

More recent efforts, however, have shown that the core vestibular network is structurally^15^ and functionally^16^ interconnected with areas subserving visuospatial processing, arousal and attentional modulation (e.g., anterior insula^17–19^), sensory gain control, proprioceptive (supramarginal gyrus, SMG^20,21^), sensorimotor (SMG and cingulate cortex^22,23^), cognitive-perceptual^24^, and affective processes (SMG, subgenual cortex, and anterior insula^25–29^). Additionally, cerebral potentials, evoked by both naturalistic and artificial vestibular stimulation, are generated not only in the parieto-insular and temporoparietal cortex of the “core” cortical vestibular system, but also in the cerebellum, frontal-prefrontal, superior parietal, and temporal cortices, as well as the posterior fossa^30^. Vestibular function may therefore be linked with egocentric orientation and motor planning through body representation and ownership^31,32^. Additionally, visuospatial memory, navigation, motion perception and even object-based mental image transformations have also been shown to require processing by vestibular system substrates^24^, and all of these processes can be disrupted in cases of vestibular dysfunction. Indeed, vestibular functioning has been shown to involve the integration of multiple sensory modalities^33^, and syndromes such as persistent postural-perceptual dizziness (PPPD) and post-concussive vestibular dysfunction (PCVD) have been associated with maladaptive visual and visuospatial processing^34–37^. Furthermore, vestibular manipulation elicits affective responses^38,39^ and vestibular dysfunction may induce or exacerbate anxiety, depression, and other affective conditions^33,40^ or, conversely, can be modulated by affective conditions such as state and trait anxiety^41^. Neuroimaging research which incorporates not only cortical areas but also structures in the basal ganglia, thalamus, and posterior fossa would therefore contribute considerably to vestibular systems neuroscience and patient care. Large neuroimaging consortia comprised of datasets from both normative adults and adolescents, including the Human Connectome Project^42^, UK Biobank^43^, and Adolescent Brain Cognitive Development studies^44^, as well as those with mild traumatic brain injury, such as the Long-Term Impact of Military-Relevant Brain Injury Consortium-Chronic Effects of Neurotrauma Consortium, or LIMBIC-CENC, effort^45–47^, are available for comprehensive exploration and hypothesis testing. To date, however, no neuroimaging atlas offers the extensive coverage necessary for researchers to explore vestibular system-associated connectivity across the cortex, subcortical structures, cerebellum, and brainstem. With source validation, such coverage would also afford validation of fMRI-based vestibular research using electroencephalography (EEG) and functional near-infrared spectroscopy (fNIRS), which, while limited in spatial resolution relative to fMRI and generally constrained to cortical areas, do allow naturalistic head movements in experimental contexts^48^.

The present paper attempts to address this omission by provisioning a compilation of volumetric brain atlases in MNI stereotactic space which, in composite, comprises the entirety of the brain. It is hoped that further development and refinement of this compilation will be supported by the neuroimaging community; recent research, for example, indicates that the OP2 region may consist of functional^49^ and structural^15^ subdivisions, such as the posterior insular complex, which exhibit distinct connectivity patterns, and these subdivisions may need to be included in future versions of the compilation. Thus, the present version (denoted Eagle-449 to indicate that it includes 449 regions of interest) represents an initial effort toward providing an important resource for research into vestibular function and dysfunction and the expanding list of regions functionally associated with the vestibular system.

## Methods

### Generation of the vestibular atlas

**Fig. 1:**
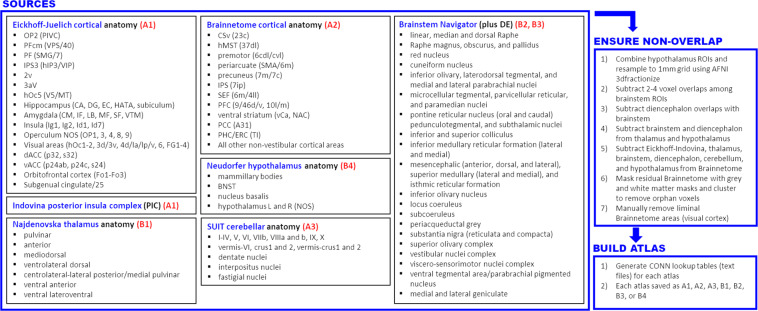
Depiction of the compiled atlas-generation process from source atlas data. Sources (largest panel) included the Eickhoff-Juelich cortical parcellation in addition to left- and right-hemisphere posterior insular complex (PIC) regions of interest (ROIs) provided by Indovina *et al*., which were incorporated into atlas A1; the Diedrichsen-SUIT anatomical cerebellum parcellation, incorporated into atlas A3; an anatomical thalamus parcellation by Najdenovska *et al*., incorporated into atlas B1; an anatomical hypothalamus parcellation by Neudorfer *et al*., incorporated into atlas B4; and brainstem and diencephalon ROIs provided as part of the Brainstem Navigator atlas and incorporated as atlases B2 and B3, respectively (see text for source references). Cortical areas not included in atlas A1 were incorporated from the Fan Brainnetome atlas as atlas A2. In all cases, source ROIs were aligned to the 1 mm isometric MNI152 T1 template and adjusted for overlaps, as detailed in the text; additionally, some hypothalamic ROIs were merged and downsampled from the source 0.5 mm isometric to final 1 mm isometric grid. All T1-registered source ROIs were masked against cerebrospinal fluid and white matter templates, and isolated voxel clusters were edited manually and via clustering (*Ensure non-overlap* panel). Finally, lookup tables were generated for each of the final seven atlases for import into the CONN Toolbox (*Build atlas* panel).

Six parcellations were leveraged for this compilation, including the Eickhoff Anatomy atlas, which is a primary component of the JuBrain/SPM Anatomy Toolbox (https://github.com/inm7/jubrain-anatomy-toolbox)^50–52^; the SUIT anatomical cerebellar parcellation by Diedrichsen *et al*. (https://github.com/DiedrichsenLab/cerebellar_atlases/tree/master/Diedrichsen_2009)^53^; anatomical parcellations of the thalamus^54^ and hypothalamus^55^ by Najdenovska *et al*.^56^ and Neudorfer *et al*.^57^ respectively; and divisions of the brainstem and diencephalon provided as part of the Brainstem Navigator template^58–64^. All cortical areas not included in the Eickhoff parcellation were obtained from the Brainnetome atlas (https://atlas.brainnetome.org) by Fan *et al*.^65–69^. All atlases were resampled to MNI152 stereotactic space, if necessary, registered to the 1 mm isometric MNI152 T1 template (version 2009c) using 3-dof linear registration, inspected and corrected for overlapping voxels, and masked against the MNI152 white matter and cerebrospinal fluid templates. “Orphan” voxels, defined as very small voxel clusters which were not contiguous with a larger cluster in-plane or within adjacent slices, were then removed using the AFNI program 3dmerge^70^, which performed voxel clustering using a maximum inter-voxel connection radius of 1.7 mm. Finally, “liminal” voxels, defined as contiguous but thin edges of 1–4 voxel thickness, and which were usually the result of remnant ROI edges, often in occipital cortex, following subtraction of other atlases from the Brainnetome parcellation, were manually removed by editing in AFNI (Fig. 1).

Several ROIs identified as “vestibular” by Eickhoff^6,7^, zu Eulenberg^8,9^, and others^14,15,71,72^, including OP2/PIVC + , the posterior insular complex (PIC), visual cingulate (CSv), somatosensory areas 2 v and 3aV, premotor area 6, and inferior parietal areas IPS3, PF, and PFcm, as well as cortical and subcortical structures subserving “multimodal vestibular” or “extended vestibular” areas which are responsive to vestibular stimulation but perform non-vestibular-specific functions, such as spatial localization and recall, visuo-vestibular processing, integration of somatosensory and motor information, and representation of peri-personal space, including the medial temporal lobe, insula, precuneus, and temporal lobe^15,16^, were well-represented in the Eickhoff-Indovina or Brainnetome parcellations. These are presented in Table 1.

**Table 1:** Regions of interest (ROIs) identified as “vestibular” or “extended vestibular” (*Extd. vestibular*) atlas number (*ROI ID*), label and label numbers within the atlas, and number of voxels within the ROI.

	ROI	ROI Label	Atlas	ROI ID	Voxels
**Vestibular**	L posterior insular complex	PIC L	A1	1	1713
	R posterior insular complex	PIC R	A1	2	1844
	L inferior parietal/supramarginal area PFcm	IPL PFcm L	A1	25	2984
	R inferior parietal/supramarginal area PFcm	IPL PFcm R	A1	75	1841
	L inferior parietal area PF/40/anterior supramarginal gyrus	IPL PF L	A1	26	4537
	R inferior parietal area PF/40/anterior supramarginal gyrus	IPL PF R	A1	76	6161
	L parietal area OP2 (parieto-insular vestibular cortex, PIVC)	OP2 L	A1	31	519
	R parietal area OP2 (parieto-insular vestibular cortex, PIVC)	OP2 R	A1	81	812
	L dorsolateral middle temporal area 37/hMST	A37dl L	A2	85	4660
	R dorsolateral middle temporal area 37/hMST	A37dl R	A2	86	5594
	L vestibular nuclei complex	VestibN L	B2	61	201
	R vestibular nuclei complex	VestibN R	B2	62	188
	L caudal cingulate area 23 (cingulate sulcus visual, CSv)	A23c L	A2	185	4731
	R caudal cingulate area 23 (cingulate sulcus visual, CSv)	A23c R	A2	186	4401
**Extd. vestibular**	L hIPS3/hIP3/VIP	AIPS IP3 L	A1	3	3606
	R hIPS3/hIP3/VIP	AIPS IP3 R	A1	53	3842
	L primary somatosensory area 2	PSC 2 L	A1	36	5186
	R primary somatosensory area 2	PSC 2 R	A1	86	5563
	L primary somatosensory area 3a	PSC 3a L	A1	37	2135
	R primary somatosensory area 3a	PSC 3a R	A1	87	1457
	L visual area V5/MT	Vis hOc5 L	A1	51	674
	R visual area V5/MT	Vis hOc5 R	A1	101	710
	L intraparietal/superior parietal area 7 (hIP3) (Brainnetome)	A7ip L	A2	133	2482
	R intraparietal/superior parietal area 7 (hIP3) (Brainnetome)	A7ip R	A2	134	2371
	L postcentral area 2 (Brainnetome)	A2 L	A2	159	1259
	R postcentral area 2 (Brainnetome)	A2 R	A2	160	2961

Abbreviations: *L*, left hemisphere; *R*, right hemisphere.

**Fig. 2:**
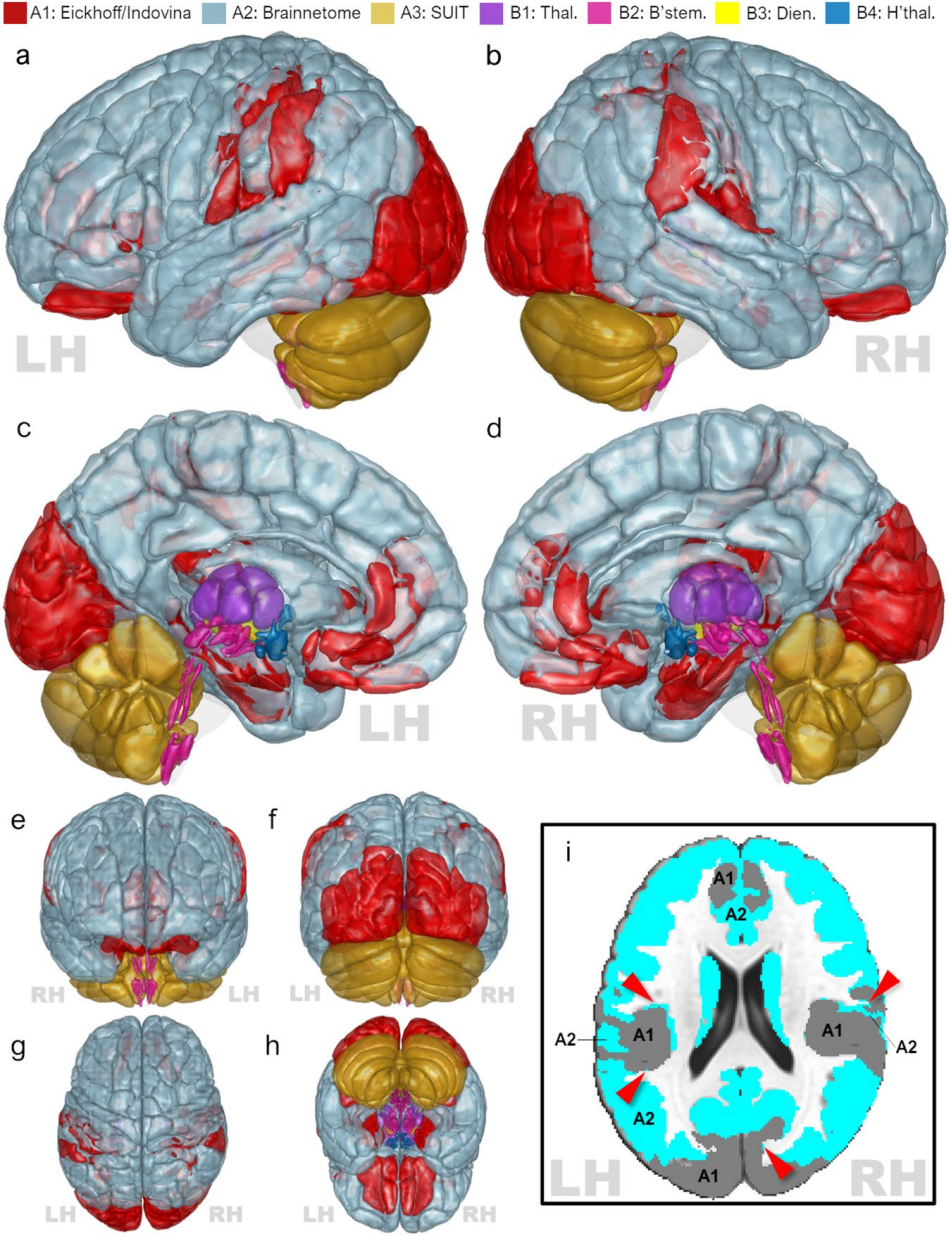
Rendering of the composite atlas. Regions of interest (ROIs) from each of the seven constitutive atlases are overlaid on the 1 mm isometric MNI152 T1 template and rendered at 50% opacity. Note that ROI extents are approximate due to the necessity of vertex smoothing operations for rendering. (***a***) Left view; (***b***) right view; (***c***) left medial view; (***d***) right medial view; (***e***) anterior view; (***f***) posterior view; (***g***) superior view; (***h***) inferior view. Inset (***i***) depicts a representative axial section (*z* = +21 mm) demonstrating that some Brainnetome ROIs (atlas *A2*, *blue*) are remnants of anatomically-defined areas which are generally, but not entirely, included in the Eickhoff/Indovina-derived atlas (atlas *A1*, *grey*). These ROIs are often liminal to, or interdigitated with, the larger atlas A1 ROIs (*red arrows*). There is, therefore, some overlap in ROI names between the two atlases, and users are discouraged from leveraging atlas A2 without inclusion of atlas A1. *LH* and *RH* indicate left and right hemisphere, respectively. Abbreviations: *Thal*., adapted from anatomical thalamus parcellation by Najdenovska *et al*.; *B’stem*, *Dien*., brainstem and diencephalon ROIs adapted from Brainstem Navigator; *H’thal*., adapted from anatomical hypothalamus parcellation by Neudorfer *et al*.

Additional preprocessing details for each parcellation are provided below. For a comprehensive list of all ROIs obtained from each atlas, as well as atlas labels, ROI values, and ROI size, refer to **Supplementary Tables – Atlas Labels**. All preprocessed parcellations, ROI label files (also known as lookup tables or LUTs), and preprocessing code have been made available on the Neuroimaging Informatics Tools and Resources Collaboratory Resources Registry (NITRC repository)^73^. The compiled atlas is presented as Fig. 2.

### Cortical parcellation

#### Eickhoff-Juelich anatomical cortical parcellation (*Atlas A*1)

The Eickhoff-Juelich (JuBrain) cytoarchitectonic parcellation is comprised of several known vestibular ROIs, including divisions of the parietal operculum, such as OP2 and secondary somatosensory cortex^6,7,74^, primary somatosensory cortex, including area 3a^75,76^, visual area MT/V5^77^, superior parietal cortex, including area 7^78,79^, and inferior parietal cortex, including areas PF and PFcm^80,81^. A number of ROIs encompassing the hippocampus^82^, insula^83^, amygdala^82^, cingulum^84^, orbitofrontal cortex^85^, and visual areas^86–90^ were also included from this parcellation. 102 JuBrain atlas ROIs, along with left- and right-hemisphere posterior insular complex (PIC), derived from the disjunction of the Brainnetome atlas BA40rv and Eickhoff atlas OP2 ROIs and provided by Indovina *et al*.^15^, were incorporated as *Atlas A1*. To create Atlas A1, the left (L) and right (R) PIC ROIs, as well as SUIT cerebellar (Atlas A3), thalamic (Atlas B1), and brainstem and diencephalon ROIs (atlases B2 and B3) were subtracted from other JuBrain atlas regions.

#### Brainnetome structural-functional cortical parcellation (*Atlas A*2)

The JuBrain parcellation does not comprise the entire cerebral cortex, and some areas commonly associated with vestibular processing, including hMST and visual cingulate (caudal Brodmann area 23), are not well-defined. Consequently, all cortical ROIs not defined in Atlas A1 were generated from the Brainnetome atlas, which is premised on the functional and structural connectivity of cortical regions, as *Atlas A2*. To avoid overlaps with other parcellations, a binary mask comprised of atlases A1, A3 (cerebellar), B1 (thalamic), B2 (brainstem), and B3 (diencephalon) was subtracted from the Brainnetome parcellation. As a result of this subtraction, some ROIs conventionally included in the Brainnetome parcellation, including occipitopolar cortex (OPC), medioventral (cLinG) and lateral occipital cortex (iOccG), L lateral amygdala (lAmygL), and R entorhinal area 28/34 (A28/34), contain zero voxels in the final Atlas A2. However, these regions are indexed in the LUTs for comprehensiveness, for a total 229 Atlas A2 ROIs.

The Brainnetome parcellation does not contain cerebellar ROIs, but does define several ROIs from subcortical structures, including cingulate, caudate, putamen, globus pallidus, and nucleus accumbens, and these structures are also included in Atlas A2. Additionally, some larger cortical areas, such as superior parietal area hIPS3 and postcentral area 2, are more restrictively defined in the JuBrain/A1 atlas, and portions of these areas which are contiguous with, but do not overlap with, A1 areas are also included in Atlas A2. Finally, Atlas A2 contains liminal remnants of other cortical regions which were not defined as part of other atlases, but were too large and contiguous to be considered “orphan” voxel clusters. These remnants are typically found in the occipital lobe in the vicinity of the parieto-occipital sulcus.

### Cerebellar parcellation

#### Diedrichsen-SUIT anatomical parcellation (*Atlas A*3)

The SUIT cerebellar atlas is premised on anatomical delineations of cerebellar structures and is provided as part of the SUIT Toolbox. It was incorporated into this compilation, without modification, as *Atlas A3*, and is comprised of 34 ROIs. ROIs included in the SUIT parcellation were subtracted from other atlases, as noted elsewhere in this section.

### Subcortical and brainstem structures

#### Najdenovska anatomical thalamus parcellation (*Atlas B*1)

Many atlases include a delineation of the thalamus which is based on the functional connectivity analyses by Behrens *et al*.^91,92^. Our present atlas compilation, however, is primarily based on anatomical delineation of cerebral structures, and, for consistency, we opted to include the thalamic parcellation by Najdenovska *et al*.^56^ as *Atlas B1*, comprised of 14 ROIs. Brainstem and diencephalon ROIs (atlases B2 and B3) overlapped with the edges of some of these thalamic areas by approximately 1–3 voxels, and were subtracted from Atlas B1 accordingly. A mask comprised of ROIs from Atlas B1 was also subtracted from atlases A1 and A2, as previously noted, as well as from Atlas B4 (hypothalamus). Lateral and medial geniculate nuclei (LGN, MGN) were included with diencephalic regions (Atlas B3).

#### Brainstem Navigator (brainstem and diencephalon: Atlases B2 and B3)

Brainstem and diencephalon ROIs were incorporated into this atlas compilation as Atlases B2 and B3, respectively. Some minor overlaps of 1–2 voxels were noted among these ROIs; accordingly, each pair of ROIs was assessed for voxel overlap, and overlapping voxels were retained from the smaller, and subtracted from the larger, of the pair. A mask of the Atlas B2 and B3 ROIs was created following this procedure and subtracted from atlases A1 and B1, as previously noted, as well as from Atlas B4 (hypothalamus). The final B2 atlas was comprised of 66 ROIs, and the B3 atlas of 6 ROIs, including L and R LGN and MGN and L and R subthalamic nuclei. Notably, the vestibular nuclei complex, an important component of the vestibular system, is included in Atlas B2 (Table 1).

#### Neudorfer anatomical hypothalamus parcellation (*Atlas B*4)

The detailed hypothalamic parcellation by Neudorfer and colleagues^57^ is originally defined in 0.5 mm isometric space and required additional processing for inclusion in the 1 mm compilation. As part of this process, larger ROIs, such as the L and R mamillary bodies, bed nuclei of the stria terminali, and L and R nucleus basalis were retained; however, the final 1 mm resolution, relative to the size of several other nuclei of interest, including the medial preoptic, paraventricular, periventricular, dorsal periventricular, ventromedial, dorsomedial, supraoptic, suprachiasmatic, tuberomammillary, and arcuate nuclei, as well as the lateral, anterior, and posterior hypothalamus, necessitated their merger into L and R “hypothalamus, not otherwise specified (NOS)” ROIs. Downsampling to the 1 mm isometric grid was performed with the AFNI program 3dfractionize; various 3dfractionize clipping levels (percent voxel occupation) from 0.1 (10%) to 0.8 (80%) were assessed, and a clipping level of 40% ultimately selected, as this yielded ROI borders which most closely matched those in the original, 0.5 mm isometric grid. Minor overlaps between the Neudorfer hypothalamus, Brainstem Navigator, and Najdenovska thalamus ROIs were resolved by subtraction of binary masks representing atlases B1, B2, and B3 from the downsampled ROIs. Eight ROIs remained after this subtraction and are included in the compilation as *Atlas B4*. A partial view of the original Neudorfer *et al*. and final Atlas B4 ROIs, following downsampling and subtraction of brainstem and diencephalon ROIs (Atlas B2 and B3), is provided as Supplementary Figure 2.

## Data Records

The Eagle-449 atlas compilation has been made available on NITRC^73^, under an open/attribution license, in the form of Gzipped, NIfTI-format volumetric datasets for atlases a1-a3 and b1-b4; text-format lookup tables (LUTs) for each atlas based on assigned ROI value; a spreadsheet containing ROI values, abbreviations, full labels, ROI group (e.g., *somatomotor, orbitofrontal, cerebellum*); and suggested ROI labels and ROI ordering for use with the CONN Toolbox^73^. The present version, v449, resides in the *EAGLE449* folder on the associated GitHub repository, and it is expected that future iterations of the atlas compilation, which will be based on additional contributions from vestibular neuroimaging researchers, will be placed in separate subfolders in the same repository.

## Technical Validation

### Participants and MRI acquisitions

The present study evaluated rsfMRI data from a total 821 datasets from young, healthy adult subjects (402 male, 419 female) included in the HCP S500 (n = 269), S900 (n = 350), and S1200 (n = 202) public data releases. Additional information on HCP inclusion and exclusion criteria may be found in Van Essen^42^. One rsfMRI acquisition, acquired using a Siemens Skyra 3 T MRI (1200 frames at TR = 720msec, 72 slices, TE = 33.1msec, FA = 52°, 2 mm isometric voxels, 208 × 180 mm FOV)^93^, previously denoised by the HCP using the FIX denoising pipeline and normalized to the standard MNI152 brain template (REST1_LR_hp2000_clean), as well as a preprocessed T1-weighted anatomical image, was obtained for each participant. The FIX pipeline includes application of a highpass filter at 2000 sec, motion regression, and regression of noise components identified via independent components analysis^94^. Only data preprocessed with the most recent HCP preprocessing workflow, version r227, were included. Subject ages ranged from 22 to 36 years (see Table 2).

**Table 2:** Age and sex of Human Connectome Project (HCP) participants leveraged for the present study.

	22–25 yrs	26–30 yrs	31–35 yrs	≥36 yrs	Total
Female	69	187	155	8	419
Male	124	179	97	2	402
Total	193	366	252	10	821

### Data preprocessing

Given the denoising and normalization already applied to the subject T1 and resting state data as part of the FIX-denoising process, the current workflow, performed in the CONN Toolbox version 21b, was limited to minimal preprocessing standards and consisted of tissue segmentation to obtain mean white matter and CSF signal, minimal correction or residual subject motion and identification of framewise outliers, ordinary least-squares regression of white matter, CSF, and motion noise sources, and smoothing of residual data using a 4 mm FWHM Gaussian kernel as well as an 8–250 mHz bandpass. Quality assurance results are provided as Supplementary Figure 1**– Quality Control**.

### Functional connectivity analysis

To test the atlas compilation, semipartial correlations were computed, using the CONN Toolbox, between the right (R) OP2, R PIC, and all 447 remaining ROIs from the seven component parcellations. The semipartial correlation coefficient, or SPCC, between ROI pairs controls for other ROIs’ mediating effects on those pairs, and thus represents an estimate of the “direct” or “effective” connectivity between them^95–97^. For this analysis, the Fisher-transformed SPCC, *Z*(*i, j*), was computed for each pair of ROIs using the ROI timeseries *R*_*i*_ (*t*) and the matrix *β* of multivariate regression coefficients:

$${R}_{j}\left(t\right)=\sum _{i\ne j}\beta \left(i,j\right){R}_{i}\left(t\right)+{\epsilon }_{j}(t)$$

where *β*(*i, j*) is estimated by ordinary least squares:

$$\beta (i,j)|\mathop{\min }\limits_{\beta (i,j)}\int {\epsilon }^{2}(t)dt,$$

and the normalized SPCC,

$$Z\left(i,j\right){=\tanh }^{-1}\left(\beta (i,j)\sqrt{c}\right),$$

Is computed as

$$c=\frac{\int {\widetilde{R}}^{2}\left(t\right)\;dt}{\int {R}_{j}^{2}\left(t\right)\;dt}$$

The matrix **Z** of all SPCCs was then subjected to a one-sample *t-*test and a “threshold-free cluster enhancement” (TFCE) score^98^ computed for each connection and for groups of neighboring connections (subgraphs or “clusters”) from the resulting matrix of *t-*statistics. Hypothesis testing proceeded by comparing the *t-*value for each connection with an estimate of a TFCE “extent” parameter at various thresholds and comparing the results with an expected null distribution of TFCE values estimated using 1000 permutations of the original data. This procedure yielded a “peak-level” familywise error rate (FWE)-corrected p-value, representing the likelihood of detecting one or more connections with the observed TFCE value or larger over all connections in **Z**. “Peak-level” uncorrected and false discovery rate (FDR)-corrected p-values were also estimated for each local extremum in the TFCE score matrix, representing the likelihood of observing its TFCE score, or larger, in at least one other randomly-selected extremum in the TFCE score matrix, or the expected proportion of false discoveries for that extremum’s TFCE score or greater^97,99^. For hypothesis testing, the current TFCE results were corrected for multiple comparisons at p(FWE) ≤0.05.

The CONN Toolbox computes TFCE using an exact integration method. Note that the definition of a “cluster” of connections for TFCE is partly dependent on ROI sorting, and this sorting algorithm may be implemented manually or through hierarchical clustering based on anatomical proximity or functional similarity^100^. For the present (exploratory) purposes, ROIs were sorted by anatomical or functional domain, including *vestibular* or *extended vestibular* based on prior publications^14,15^, or *orbitofrontal-prefrontal*, *frontal, somatomotor, somatosensory, visual, superior* or *inferior parietal, superior* or *middle-temporal*, *medial temporal, insular*, and *cingulate*. FWE, FDR, and uncorrected p-values for connection clusters and individual connections did not differ substantially between this manual sorting and the default CONN hierarchical algorithm; other algorithms may, however, be preferable^101^.

### Results

**Fig. 3:**
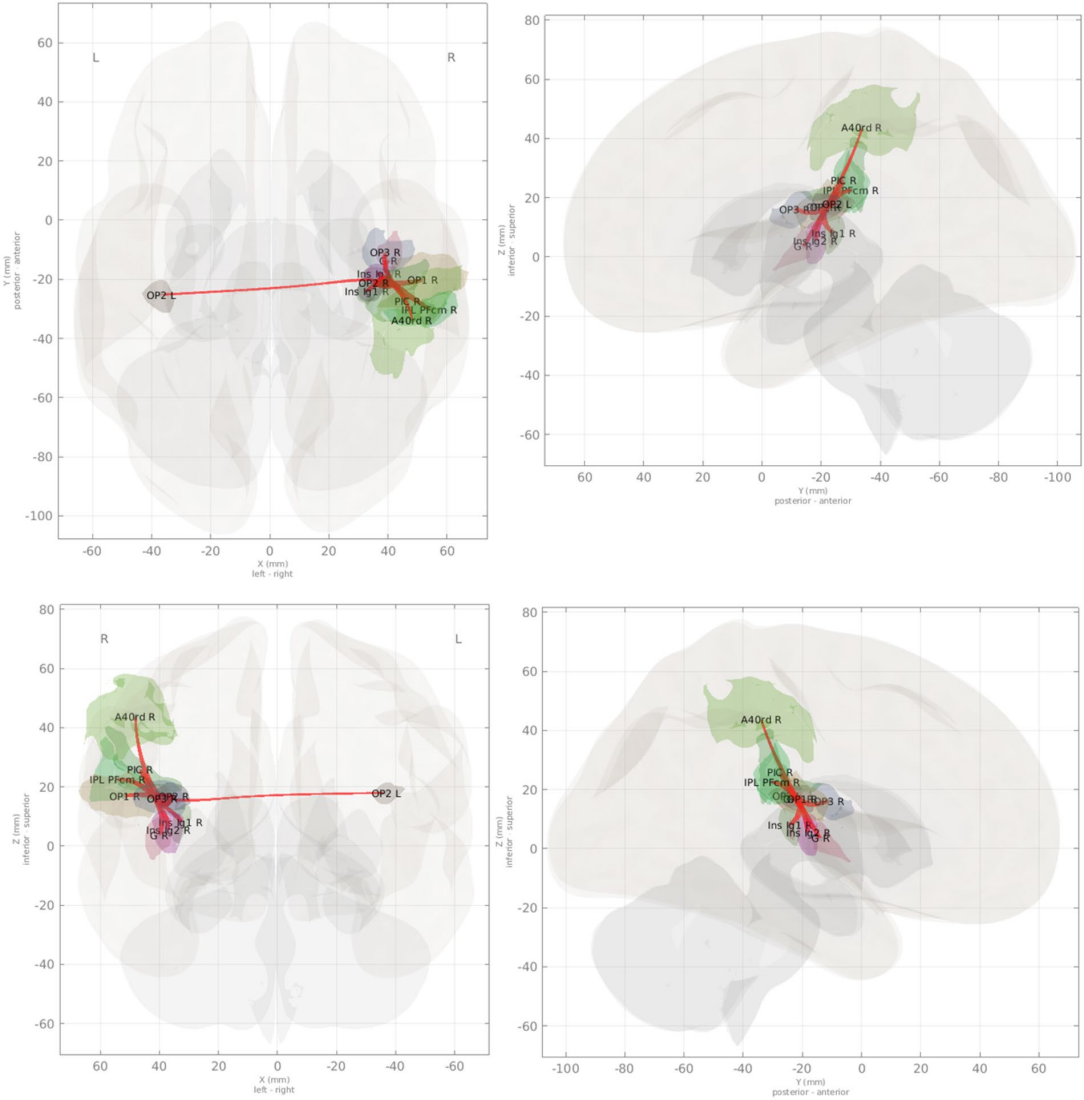
Connections of right-hemisphere parieto-insular region OP2, derived from semi-partial correlations with the R OP2 seed voxel signal after preprocessing and denoising. Connections in this figure are adjusted for multiple comparisons correction using threshold-free cluster enhancement (TFCE) with a familywise error rate *p* ≤ 0.05 (see *Technical Validation – Functional connectivity analysis* section of the text). Compare with Indovina *et al*.^15^.

**Fig. 4:**
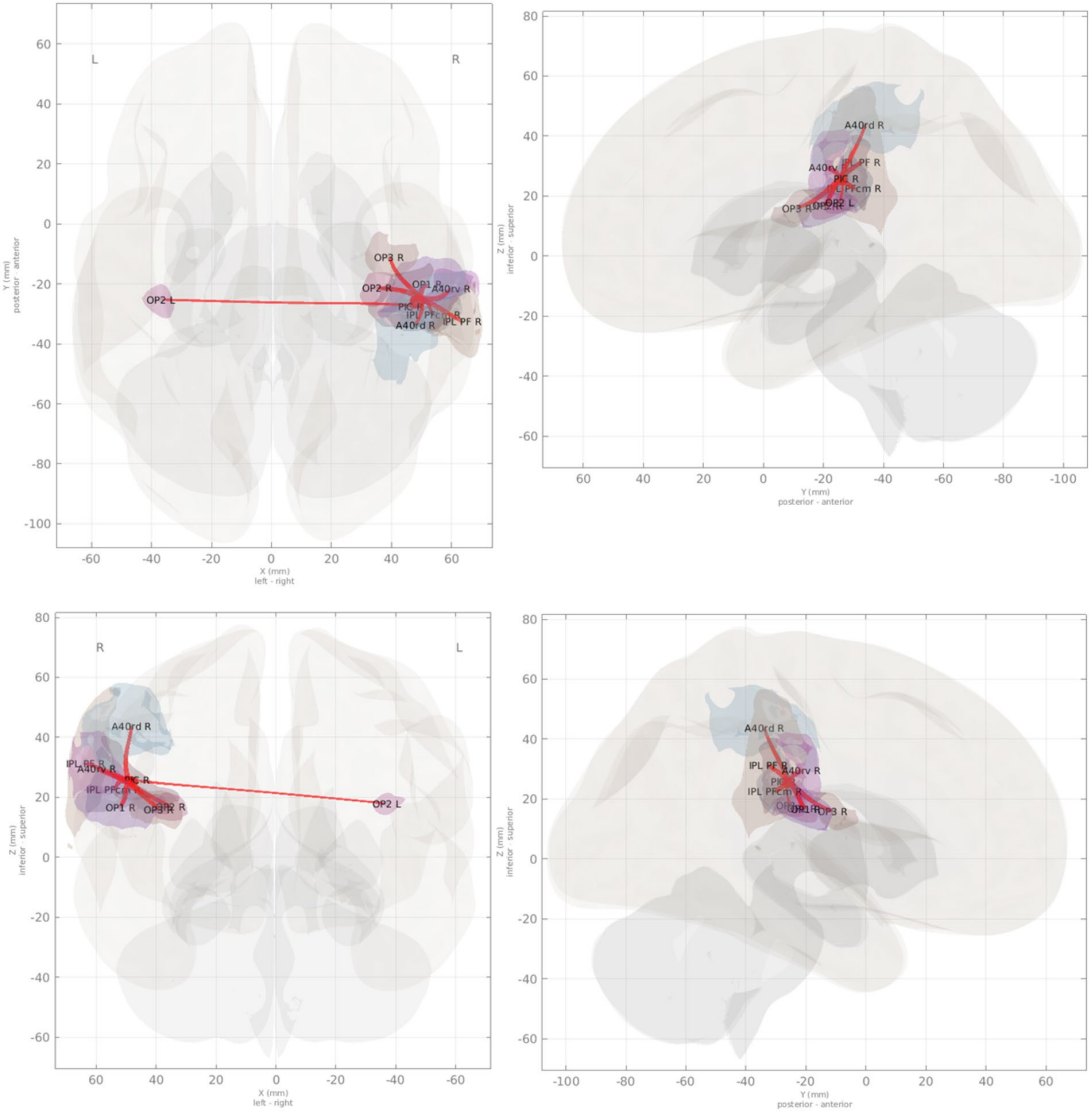
Connections of right-hemisphere parieto-insular region PIC, derived from semi-partial correlations with the R PIC seed voxel signal after preprocessing and denoising. Connections in this figure are adjusted for multiple comparisons correction using threshold-free cluster enhancement (TFCE) with a familywise error rate *p* ≤ 0.05 (see *Technical Validation – Functional connectivity analysis* section of the text). Compare with Indovina *et al*.^15^.

Semipartial correlations, corrected for multiple comparisons using TFCE, are shown for the R OP2 and R PIC seed ROIs in Fig. 3, 4, respectively, and results provided for first-order connections to these ROIs are presented in Table 3. R OP2 connections in this normative cohort included R parietal area PFcm, OP1, OP3, insular areas G, Ig1, and Ig2, PIC, and rostrodorsal area 40 (40rd/area PFt), as well as L OP2. Connections with R PIC included R PFcm, PF, OP1, OP3, OP2, rostroventral area 40 (area PFop), and rostrodorsal area 40/PFt, as well as L OP2. Note that, although TFCEs for the R OP2:R PFcm, R OP2:R 40rd/PFt, R PIC:40rd/PFt, and R PIC-L OP2 parent clusters were statistically significant at *p*(*FWE*)≤0.05 and *p*(*FDR*)≤0.05, these associations did not survive multiple comparisons correction at the connection level. These results compare favorably with the structural findings of Indovina *et al*.^15^ except that no connection between R PIC and L motor area 4a was observed in the current results, but were reported by Indovina *et al*., and a connection between R OP2 and ipsilateral PFt was observed in the current results, but was not reported by Indovina *et al*. Additionally, a suprathreshold number of streamlines originating from, or terminating in, R OP2 or R PIC, were observed in the latter study which were not supported, in terms of functional connectivity, here. Neither R OP2 nor R PIC exhibited connectivity with thalamic, brainstem, or cerebellar nuclei. In both cases, however, connectivity was predominantly localized to the right hemisphere, and characterized by homotopic connections with the seed ROIs. All semipartial correlations were positive, indicating in-phase relationships between the seed and target ROIs.

**Table 3:** First-order connections of right (R) OP2 and R PIC derived from semipartial correlations across all 449 pairs of ROIs in the composite atlas and corrected for multiple comparisons using TFCE.

R OP2	R PIC	Connection	t(820)	p(FDR)	p(FWE)
		Cluster 75/690: TFCE 3174.31, p(unc) <0.0001, p(FDR) <0.0001, p(FWE) <0.0001			
	■	[EI] PIC R: [EI] IPL PFcm R	21.55	<0.0001	<0.0001
	■	[EI] PIC R: [EI] IPL PF R	6.55	<0.0001	<0.0001
■		[EI] OP2 R: [EI] IPL PFcm R	1.79	0.0741	0.5974
		Cluster 164/690: TFCE 1136.96, p(unc) <0.0001, p(FDR) <0.0001, p(FWE) <0.0001			
	■	[EI] PIC R: [EI] OP1 R	15.16	<0.0001	<0.0001
■		[EI] OP2 R: [EI] OP1 R	6.77	<0.0001	<0.0001
		Cluster 446/690: TFCE 118.4, p(unc) <0.0001, p(FDR) <0.0001, p(FWE) <0.0001			
■		[EI] OP2 R: [EI] OP3 R	7.28	<0.0001	<0.0001
	■	[EI] PIC R: [EI] OP3 R	3.64	0.0003	0.0133
		Cluster 475/690: TFCE 98.7, p(unc) <0.0001, p(FDR) <0.0001, p(FWE) <0.0001			
■		[EI] OP2 R: [EI] Ins Ig1 R	6.59	<0.0001	<0.0001
		Cluster 504/690: TFCE 81.67, p(unc) <0.0001, p(FDR) <0.0001, p(FWE) <0.0001			
■		[EI] OP2 L: [EI] OP2 R	5.42	<0.0001	<0.0001
	■	[EI] OP2 L: [EI] PIC R	2.51	0.0122	0.2552
		Cluster 571/690: TFCE 55.68, p(unc) <0.0001, p(FDR) <0.0001, p(FWE) <0.0001			
■		[EI] OP2 R: [EI] Ins Ig2 R	4.65	<0.0001	0.0002
		Cluster 583/690: TFCE 53, p(unc) <0.0001, p(FDR) <0.0001, p(FWE) = 0.001			
■	■	[EI] OP2 R: [EI] PIC R	4.3	<0.0001	0.0011
		Cluster 607/690: TFCE 48.52, p(unc) <0.0001, p(FDR) <0.0001, p(FWE) = 0.002			
■		[EI] OP2 R: [BN] G R	5.35	<0.0001	<0.0001
		Cluster 650/690: TFCE 42.67, p(unc) <0.0001, p(FDR) <0.0001, p(FWE) = 0.01			
	■	[EI] PIC R: [BN] A40rv R	5.15	<0.0001	<0.0001
■		[EI] OP2 R: [BN] A40rd R	1.63	0.1041	0.6648
	■	[EI] PIC R: [BN] A40rd R	0.78	0.4353	0.9016

ROI abbreviations are listed in Supplementary Table 1. A report containing statistics for connections of all vestibular areas (as listed in Table 1) is provided as Supplementary Table 2. Other abbreviations: *FDR*, false discovery rate correction; *FWE*, familywise error rate correction; *unc*, uncorrected.

Statistics for all extant connections following TFCE correction are also provided as Supplementary Table 2. Several interesting connections were observed between conventional vestibular regions and areas not typically included in the “core” vestibular network, including R somatosensory area 2 and ipsilateral rostral hippocampus (connection *t* = +2.3800, *p*(*FDR*) = 0.0176, but *p*(*FWE*) = 0.3157), R area 7ip and ipsilateral laterobasal amygdala (connection *t* = +1.8700, but *p*(*FDR*) = 0.0623 and *p*(*FWE*) = 0.5625), and extensive interconnectivity between cortical vestibular areas 37 dl/hMST and 7ip and visual areas FG2, FG4, lateral-anterior and lateral-posterior area hOc4, and between hOc5, postcentral area 2, and regions of the cerebellum, including L crus, R V, VI, VIIb, and VIIIa. Additionally, several cortical and subcortical vestibular areas, including area PF and the vestibular nucleus complex, were functionally connected with subcortical and brainstem nuclei, such as the subcoeruleus, laterodorsal tegmental nucleus, medial parabrachial nucleus, globus pallidus, and caudal-dorsal cingulate area 24.

### Limitations of the validation process

The current version, v449, of the atlas compilation, as well as its technical validation, incurs several caveats. With respect to the compilation itself, it is important to note that the Brainnetome parcellation (Atlas A2) serves as a “catch-all” for cortical areas not included in Atlas A1, and, as previously noted, includes liminal remnants of Atlas A1 ROIs. Consequently, Atlas A2 should not be used independently of Atlas A1, and future versions of the atlas compilation may substitute other anatomically-derived cortical parcellations for, or in addition to, the Brainnetome source. Investigators are also encouraged to consider whether the Brainnetome parcellation comports with their research aims, as some cortical parcellations, including functionally-defined parcellations such as that of Gordon *et al*.^102^ may be more suitable for specific purposes. Additionally, based on recent analyses which have delineated functional subdivisions within vestibular areas, including OP2, it appears likely that these areas, as currently defined, are composites of subregions which subserve differential vestibular functions^49^.

“Validation” of the current atlas compilation was conducted by computing semi-partial correlation coefficients between right-hemisphere OP2 and PIC seeds and all other ROIs, and comparing the results to previous peer-reviewed findings concerning the connectivity of these regions^14,15^. While these qualitative comparisons were favorable in that they replicated prior results, it should be noted that there are numerous ways, in addition to semi-partial correlation, to assess RSFC. Analyses which leveraged “classical” Pearson correlations failed to resolve network structures, such as connections between vestibular nuclei and other areas, which would be expected based on prior anatomical and structural findings in humans and lower primates, and the technical validation process therefore leveraged semi-partial correlations in order to control for the influence of all other connections with the seed and target ROIs. Due to the nature of semi-partial correlation, however, removing one or more of these regions may effect a new network topology. Finally, the topologies resolved in this validation process are likely incomplete, due not only to inherent issues of multiple comparisons (which were addressed to some degree by the use of TFCE), but potentially due to suppressed effect sizes in the connectivity of the subcortical regions of HCP datasets due to MRI acquisition parameters, which have been noted as problematic by Risk and colleagues^102,103^.

## Usage Notes

The entirety of the dataset may be downloaded from GitHub (https://github.com/EmoryPcvdLab/EagleVAC) or NITRC^73^. The current version, v449, resides in the EAGLE449 folder of the GitHub repository. As the present authors did not have a role in the acquisition, processing, or distribution of the source atlases, investigators who leverage the atlas are requested to cite these original sources as described in the README file of the repository. It is also requested that investigators consider contributing to iterative refinements of the EAGLE atlas compilation, including, but not limited to, correction of errors, modifications to ROIs, and newly-derived subdivisions of existing vestibular areas documented in published, peer-reviewed research. Finally, investigators should be aware of the number of simultaneous tests (number of ROIs) relative to their sample size when leveraging these parcellations.

## Supplementary information

Supplementary Figures

Supplementary Table 1

Supplementary Table 2

## Data Availability

The BASH shell script used for merging of source atlases has also been made available on the GitHub repository as *merge_code.bash*. Investigators who wish to replicate the workflow will need to download the original source atlases and adjust file paths in the shell script to point to them. The script requires a recent version of the AFNI suite^70^.
