# Supplementary material for: Eagle-449: A volumetric, whole-brain compilation of brain atlases for vestibular functional MRI research
Source: Sci Data. 2023 Jan 14;10:29. doi: 10.1038/s41597-023-01938-1 (PMC9840609; doi:10.1038/s41597-023-01938-1)

Supplementary Figure 1 – Quality control. Quality control and assurance results comparing unsmoothed HCP resting-state data prior to the current preprocessing workflow (*i.e.,* HCP FIX-denoised only) to the same data following the preprocessing procedures (*i.e.,* HCP FIX-denoised + current preprocessing and denoising in the CONN Toolbox, version 20b). (*a*) Distribution of participant quality control measures prior to denoising: maximal mean (*MeanMotion*) and maximal framewise motion (*MaxMotion*) observed in any participant were 0.14mm and 0.18mm, respectively, and mean motion was approximately 0.02mm across all participants. Maximal mean (*MeanΔGS*) and maximal global framewise BOLD signal change (*MaxΔGS*), which are reported by the CONN Toolbox as *z*-scores, were z = 0.91 and 68.10, respectively, and mean framewise global signal change was z = 0.81 across all subjects. TRs with displacements > 0.9mm or signal changes > 5 standard deviations were flagged as outliers and excluded from further analysis. The minimum number of valid TRs (*ValidScans*) following this "scrubbing" procedure was 1054 of 1200 (87.83%), whereas an average of YYY TRs (ZZZ%) were retained across all participants. (*b*) Distribution of connectivities (Pearson *r* values) prior to (*top*) and following (*bottom*) denoising. Denoising effectively normalized connectivities and centered the mean at r = 0.00, as expected. (*c*) Functional connectivity between 1000 randomly-selected grey matter voxels as a function of distance between voxel pairs prior to (*top*) and following (*bottom*) denoising. Note that the preprocessing workflow largely eliminated correlations between intervoxel distance and connectivity for all but the closest voxel pairs. **Abbreviations**: *IQR,* interquartile range; *Qu*, quartile.

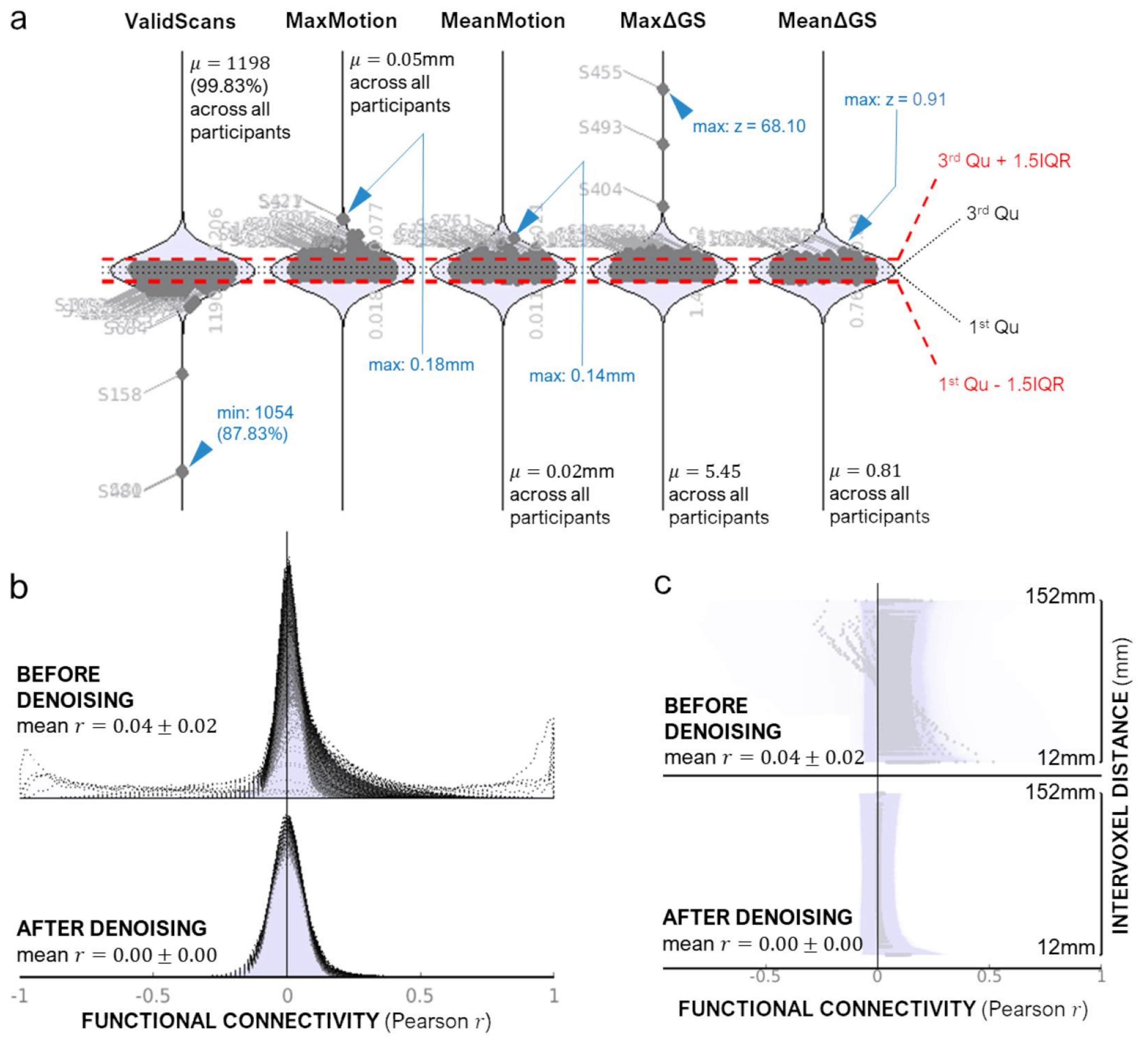

Supplementary Figure 2 – Downsampling of the hypothalamus source atlas. The hypothalamic atlas of Neudorfer et al. (source Atlas B4) required downsampling from its 0.5mm isotropic native resolution to the 1.0mm isotropic resolution of the composite atlas. Additionally, several of its regions of interest (ROIs) overlapped with those of the brainstem (Atlas B2) and diencephalon (Atlas B3) and were removed. Downsampling was conducted by a voting algorithm using the AFNI program 3dFractionize; smaller ROIs were merged into a single "hypothalamus, not otherwise specified" label, whereas larger ROIs, including the mamillary bodies, bed nucleus of the stria terminalis, and nucleus basalis, were retained. The original source atlas is shown overlaid on the 0.5mm-resolution MNI T1 brain as row (A), and the final, downsampled atlas overlaid on the 1.0mm-resolution MNI T1 brain as row (B). **Abbreviations**: *ac*, anterior commissure; *ah*, anterior hypothalamus; *arc*, arcuate nucleus; *BNST*, bed nucleus of the stria terminalis; *fnx*, fornix; *itp*, inferior thalamic peduncle; *MB*, mamillary body; *mtt*, mammillothalamic tract; *NB*, nucleus basalis; *NOS*, hypothalamus – not otherwise specified; *phn*, posterior hypothalamic nucleus; *stn*, subthalamic nucleus.

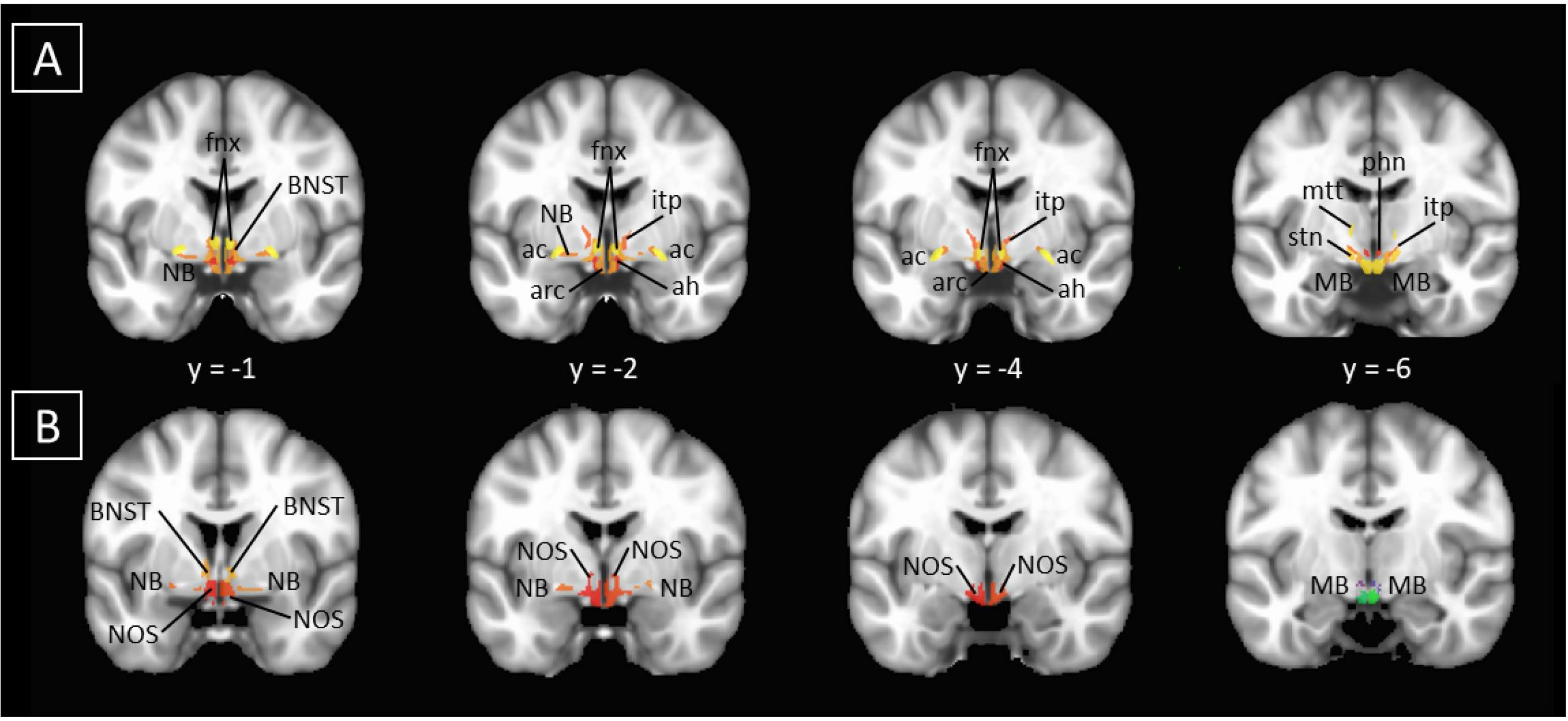

Supplement: Supplementary file 1 — Supplementary Figures [file 41597_2023_1938_MOESM1_ESM.pdf]
